# Massive Chylous Ascites in a 9-Year-Old Girl with Malrotation—A Case Report

**DOI:** 10.1055/a-2221-9682

**Published:** 2024-01-10

**Authors:** Hans Winberg, Pär Gerwins, Kristine Hagelsteen

**Affiliations:** 1 Pediatrics, Department of Clinical Sciences Lund, Lund University, Lund, Sweden; 2 Department of Pediatric Surgery, Skånes Universitetssjukhus Lund, Lund, Sweden; 3 Department of Surgical Sciences, Radiology, Uppsala University, Uppsala, Sweden

**Keywords:** malrotation, minimal invasive surgery, chylous, sealing device, Ladd's procedure

## Abstract

Malrotation leading to massive chylous ascites is rare. A 9-year-old girl was investigated for slowly increasing abdominal distension under a year. She had no vomiting, weight loss, or pain, but was bothered in social situations. Medical investigations, including ultrasound and computed tomography scans, revealed massive ascites. Laparocentesis yielded milk-colored fluid, confirmed as lymph through laboratory analysis. A complete blood count, liver function and hematologic parameters, chyle cytology, bacterial cultures, and polymerase chain reaction for tuberculosis were all within normal limits.

She was referred to a tertiary center for vascular anomalies. A dynamic contrast-enhanced magnetic resonance lymphangiography showed normal lymphatic anatomy without leakage or flow obstruction. A whole-body magnetic resonance imaging revealed a central mesenteric rotation.

She was referred to a tertiary center for pediatric surgery, where a laparoscopic Ladd's procedure was performed using a new 5 mm pediatric sealing device, along with an appendectomy using a 5 mm stapler. To derotate the bowel, fenestrations were created in compartments containing a substantial amount of chyle and ascites, resulting in the drainage of 2.4 L of fluid. She was discharged the day after surgery and has been in good health for 1 year. We present a video illustrating the Ladd's procedure steps in this patient.

## Introduction



**Video 1** Massive chylous ascites and laparoscopic Ladd's procedure.


Intestinal malrotation as the cause of massive chylous ascites is uncommon in school-age children. 1 2 3 4 Intestinal malrotation is predominately diagnosed in earlier years of life due to the obstructive effect the Ladd's band have on the intestines, blood vessels, and lymphatic system. 5 The majority is found within the first month (30%) or year (55–60%) in life, and 75% is diagnosed before 5 years of age. 5 Only a few case reports exist in the literature describing massive chylous ascites and intestinal malrotation, none for group of school age children and videos of the procedure is lacking. We present an unusual case of a 9-year-old with massive chylous ascites and intestinal malrotation. The video showcases the procedural steps of the laparoscopic Ladd's procedure and fenestration of compartments containing large amount of fluid to remove ascites ( Video 1 ).

## Case Report

A 9-year-old girl presented to the local pediatrician with progressive abdominal distension over the course of a year. She had normal vital signs, no abdominal pain, and no symptoms of vomiting or obstipation. However, she was primarily troubled by social situations at school, particularly in the dressing room. Her parents complained that she ate very little compared with her siblings and had a poor appetite. At the time of surgery, she measured 136 cm in height and 27 kg, which placed her at –0.5 standard deviations on the growth chart.

The initial medical examination and standard blood tests for malignancy and infection yielded normal test results, except for the urine sample, which showed proteinuria (2 + ) and a pathological u *rine albumin* to creatinine *ratio* of 12 (normal range < 3.0). Further investigation with an abdominal ultrasound revealed normal kidneys and spleen, but difficulties in visualizing the liver due to the massive amounts of ascites. A computed tomography (CT) indicated a normal liver and portal vein, but showed dislocated intestines on the right side of the abdomen and increased density in the area around the liver hilum, duodenum, and pancreas. The girl experienced discomfort lying down during the CT scan. Subsequent laparocentesis yielded milk-colored fluid, which was confirmed as lymph through laboratory analysis. Cytology, bacterial cultures, and polymerase chain reaction for tuberculosis all returned normal results.

**Fig. 1:**
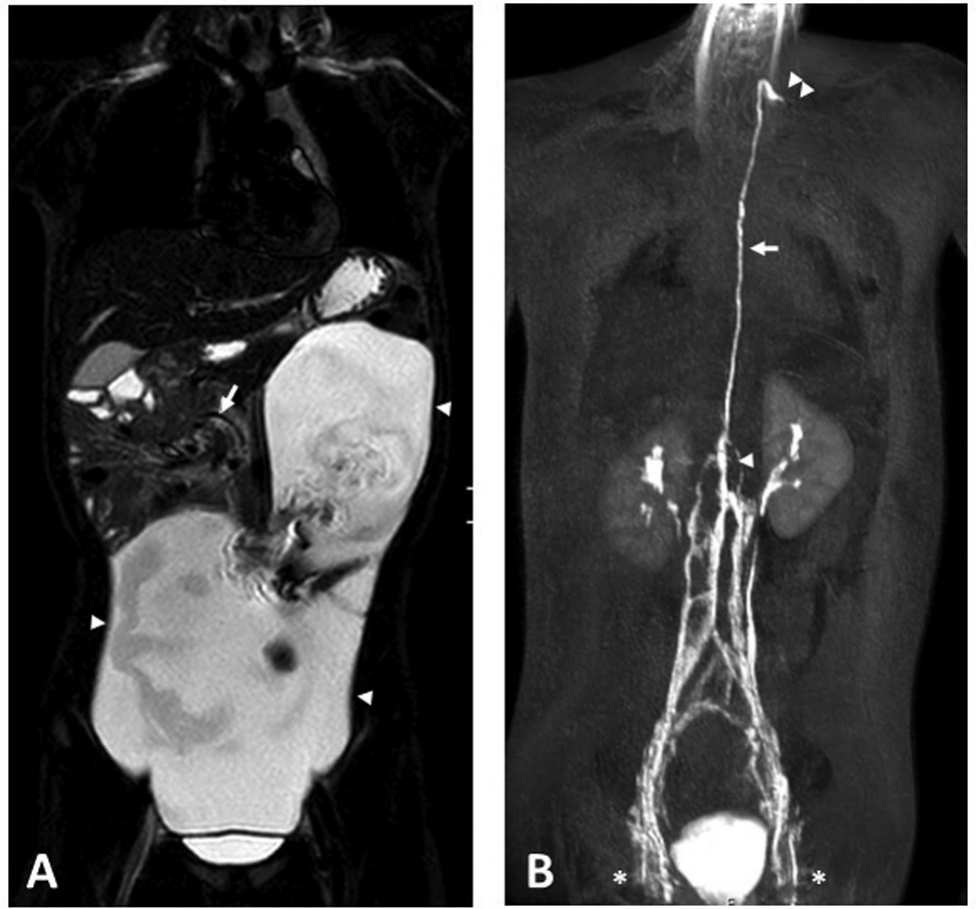
( **A** ) Magnetic resonance imaging with T2 short tau inversion recovery sequence shows large amounts of intra-abdominal fluid (arrowheads) and mesenteric rotation (arrow). ( **B** ) Magnetic resonance lymphangiography reveals normal central lymphatic vessels without signs of lymphatic leak. Gd contrast was injected into lymph nodes in the groins (*) and appear bright delineating central lymphatic vessels including cisterna chyli (arrowhead), thoracic duct (arrow), and the inflow to the subclavian vein (double arrowheads). Contrast is excreted in kidneys and bladder.

**Fig. 2:**
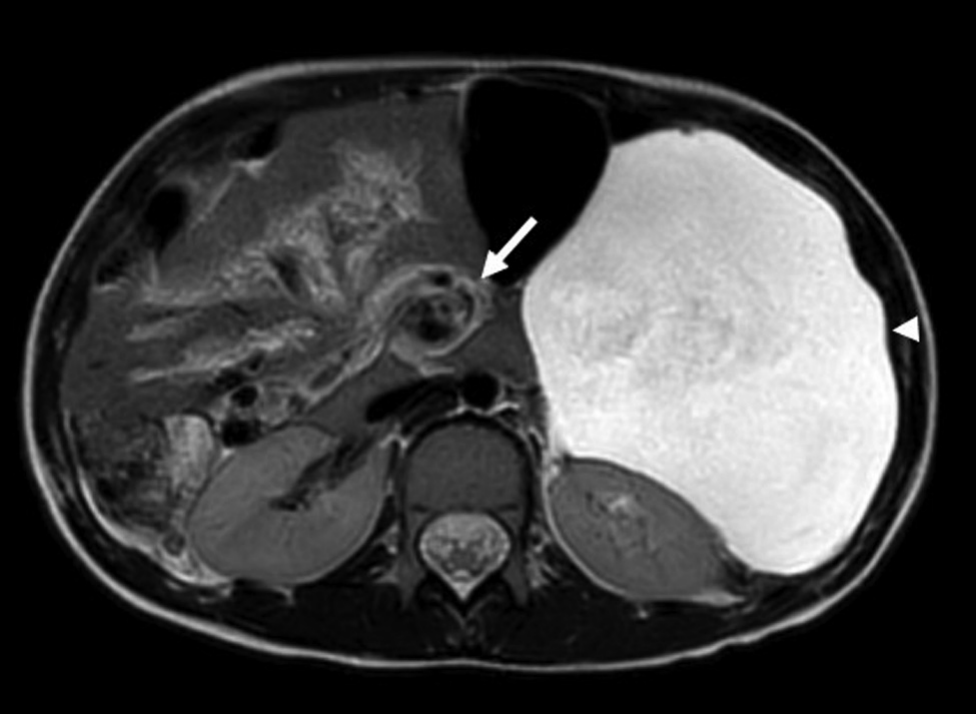
T2 magnetic resonance imaging sequence shows intra-abdominal fluid (arrowhead) and mesenteric rotation (arrow).

She was referred to a tertiary center for vascular anomalies. A dynamic contrast-enhanced magnetic resonance lymphangiography showed normal lymphatic anatomy without leakage or flow obstruction ( Fig. 1 ). A whole-body magnetic resonance imaging conducted concurrently revealed a central mesenteric rotation ( Fig. 2 ). She was referred to a tertiary center for pediatric surgery for the treatment of her intestinal malrotation with a Ladd's procedure, 10 months after her first visit to the pediatrician.

The video depicts the laparoscopic Ladd's procedure, performed using a new 5 mm pediatric sealing device for precise and controlled tissue division (Hologic, Marlborough, Massachusetts, USA). This device features dual-action jaws enabling the surgeon to perform fine adhesion dissection without the risk of thermal damage to sensitive anatomical structures ( Video 1 , available in the online version).

The Ladd's attachments were divided by mobilizing and flipping the cecum to the patients left, allowing for the visualization and division of attachments until reaching the posterior aspects of the duodenum. Ventral attachments between the duodenum and large intestine were also divided to widen the mesenteric root. The procedure proved more challenging than usual due to the extensive amount of ascites.

The ascites had become “trapped” within compartments between the intestines, exerting significant weight that made the derotation of the bowel difficult. Three separate fenestrations in these compartments were created using the sealing device to drain the chylous ascites so that we were able to run the bowel in a proximal to distal fashion and thereby derotating the intestines. These fenestrations are demonstrated in the video, with a total drainage of 2.4 L during the procedure.

Following the Ladd's band division, mesentery root widening, bowel derotation, and ensuring proper positioning of the small intestines to the right and the cecum and colon to the left, an appendectomy was performed using a 5 mm stapler (JustRight 5 mm Stapler, Hologic, United States). The patient was discharged the day after surgery. She is currently in good health, experiencing no pain, and has been eating well, showing growth consistent with her growth chart at the 1-year follow-up.

## Discussion

Chylous ascites is a rare condition, and malrotation is one of the differential diagnoses that should be considered during the investigation of these patients. Intestinal malrotation can present with various degrees of missed gut rotation and potentially different levels of compression of the mesenteric root.

In a study involving infants with chylous ascites, 9 out of 10 cases were found to have intestinal malrotation and they were effectively treated with a Ladd's procedure. 2 Speculating, based on this case and in alignment with Long et al, mild compression from the Ladd's band, which does not obstruct the superior mesentery artery, vein or the duodenum could cause lymphatic flow obstruction and leakage. 2

Performing the Ladd's surgical procedure for intestinal malrotation in patients with massive chylous ascites is challenging due to limited intraabdominal space. Additionally, the chyle becomes “trapped” in compartments, pulling the small bowel down, necessitating fenestration to evacuate the fluid to facilitate the derotation of the bowel.

## Conclusion

As illustrated in this case, massive chylous ascites due to intestinal malrotation can occur at any age. When investigating cases of massive chylous ascites, every pediatrician and pediatric surgeon should be aware of malrotation as a potential cause. Not all pediatric surgeons choose to, or are proficient in, performing a minimal invasive Ladd's procedure. Young individuals with intestinal malrotation should be referred to a pediatric tertiary center for this procedure, and we have demonstrated that it is feasible to perform the procedure laparoscopically, even in cases of massive chylous ascites.
