# Supplementary material for: Massive Chylous Ascites in a 9-Year-Old Girl with Malrotation—A Case Report
Source: European J Pediatr Surg Rep. 2024 Jan 10;12(1):e1–3. doi: 10.1055/a-2221-9682 (PMC10781518; doi:10.1055/a-2221-9682)
Supplement: Supplementary file 2 — Supplementary Material [file 10-1055-a-2221-9682-s2023030698cg.pdf]

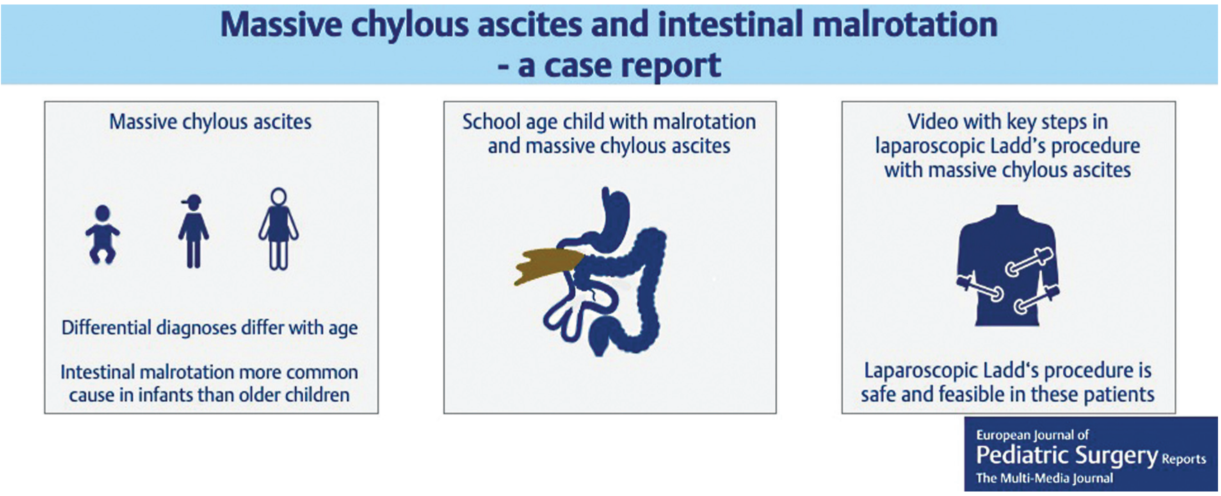

Supplementary Fig S1 Infographic.

 © 2024. The Author(s).
